# Factors associated with death outcome in patients with severe coronavirus disease-19 (COVID-19): a case-control study

**DOI:** 10.7150/ijms.46614

**Published:** 2020-05-18

**Authors:** Feng Pan, Lian Yang, Yuncheng Li, Bo Liang, Lin Li, Tianhe Ye, Lingli Li, Dehan Liu, Shan Gui, Yu Hu, Chuansheng Zheng

**Affiliations:** 1 Department of Radiology, Union Hospital, Tongji Medical College, Huazhong University of Science and Technology, Jiefang Avenue #1277, Wuhan, 430022, China.; 2 Hubei Province Key Laboratory of Molecular Imaging, Wuhan, 430022, China.; 3 Department of Otorhinolaryngology, Union Hospital, Tongji Medical College, Huazhong University of Science and Technology, 1277 Jiefang Avenue, Wuhan 430022, China.; 4 Institute of Hematology, Union Hospital, Tongji Medical College, Huazhong University of Science and Technology, 1277 Jiefang Avenue, Wuhan 430022, China.

**Keywords:** COVID-19, Critical care, Prognosis, Risk factors, Radiography

## Abstract

**Rationale**: Up to date, the exploration of clinical features in severe COVID-19 patients were mostly from the same center in Wuhan, China. The clinical data in other centers is limited. This study aims to explore the feasible parameters which could be used in clinical practice to predict the prognosis in hospitalized patients with severe coronavirus disease-19 (COVID-19).

Methods: In this case-control study, patients with severe COVID-19 in this newly established isolation center on admission between 27 January 2020 to 19 March 2020 were divided to discharge group and death event group. Clinical information was collected and analyzed for the following objectives: 1. Comparisons of basic characteristics between two groups; 2. Risk factors for death on admission using logistic regression; 3. Dynamic changes of radiographic and laboratory parameters between two groups in the course.

**Results**: 124 patients with severe COVID-19 on admission were included and divided into discharge group (n=35) and death event group (n=89). Sex, SpO2, breath rate, diastolic pressure, neutrophil, lymphocyte, C-reactive protein (CRP), procalcitonin (PCT), lactate dehydrogenase (LDH), and D-dimer were significantly correlated with death events identified using bivariate logistic regression. Further multivariate logistic regression demonstrated a significant model fitting with C-index of 0.845 (p<0.001), in which SpO2≤89%, lymphocyte≤0.64×10^9^/L, CRP>77.35mg/L, PCT>0.20μg/L, and LDH>481U/L were the independent risk factors with the ORs of 2.959, 4.015, 2.852, 3.554, and 3.185, respectively (p<0.04). In the course, persistently lower lymphocyte with higher levels of CRP, PCT, IL-6, neutrophil, LDH, D-dimer, cardiac troponin I (cTnI), brain natriuretic peptide (BNP), and increased CD4+/CD8+ T-lymphocyte ratio and were observed in death events group, while these parameters stayed stable or improved in discharge group.

**Conclusions**: On admission, the levels of SpO2, lymphocyte, CRP, PCT, and LDH could predict the prognosis of severe COVID-19 patients. Systematic inflammation with induced cardiac dysfunction was likely a primary reason for death events in severe COVID-19 except for acute respiratory distress syndrome.

## Introduction

Since the outbreak of COVID-19 in Wuhan, it spread rapidly worldwide with a severity spectrum from asymptomatic to critical illness with the overall rate of approximately 3% 1-3. Up to date, an increasing number of more than forty-thousand fatalities were reported 4. Owing to the genomic homologies of the pathogen from the coronaviruses causing the Severe Acute Respiratory Syndrome (SARS) and the Middle East Respiratory Syndrome (MERS), COVID-19 presented a similar clinical course and pathological feature 5-8. Although most of the COVID-19 patients were mild who could gradually recover after two weeks, about 15-20% of patients developed severe interstitial pneumonia 1, 8, 9. Patients with severe illness usually developed acute respiratory distress syndrome (ARDS) that requires invasive mechanical ventilation therapy in the intensive care unit (ICU), and the mortality rate was reported around 50-60% 1, 10.

To date, the clinical studies revealed different clinical and laboratory features between survivors and non-survivors, or between the non-severe and severe patients, and some prognostic risk factors were preliminarily discovered, such as age and D-dimer 6-8, 10-13. However, most of these results were from or involving the same center in Wuhan, China, at a similar period, leading to potential selection bias. Moreover, the same patients in different articles were suspected 14. This study systematically analyzed the clinical features, laboratory investigations, and radiographic estimation in a temporary isolation center in Wuhan, China, established specially for severe COVID-19 patients by an emergency directive of the Wuhan Anti-epidemic Headquarters since 27 January 2020. All severe patients had to fulfill the WHO criteria for admission to hospital for a respiratory illness, which was slightly modified and adopted by both the WHO and the Chinese National Diagnosis and Treatment Protocols to define a severe case of COVID-19 15-18. These criteria defined severe COVID-19 as fever and/or respiratory infection with positive COVID-19 nucleic acid detection, plus one of the following: respiratory rate > 30 breaths/min; severe respiratory distress; or SpO2 ≤ 93% on room air. This study examined the factors associated with mortality in patients who fulfilled these criteria of severity at the time of hospital admission. It excluded patients who at the time of admission had, according to WHO criteria, mild illness, pneumonia, or had already progressed to ARDS, sepsis, or septic shock 17. For further speculation, it tried to explore the fatal course of COVID-19.

### Methods

This study was approved by the Ethics of Committees of Union Hospital, Tongji Medical College, Huazhong University of Science and Technology (No. 2020-0026), and followed the 1964 Helsinki Declaration and its later amendments or comparable ethical standards. Only the anonymous data was collected and informed consent for this retrospective study was waived.

### Patients

The consecutive admission records were reviewed retrospectively for the period from 27 January 2020 to 19 March 2020 in this provisional center (Western Campus, Union Hospital, Tongji Medical College, Huazhong University of Science and Technology). Clinical data, including initial symptoms, past medical history, hospitalization date, treatment, and hematological and biochemical blood test results were obtained directly through the institutional electronic patient database. The inclusion criteria included: 1. Confirmed severe COVID-19 on admission as mentioned above 15-18; 2. No comorbidities which might impair the immune function, such as autoimmune disease, hematological malignancies, recent chemotherapy, etc.; 3. With definite discharge or COVID-19 related death outcome.

### Imaging Estimation

Regular bedside chest digital radiographs (DRs) were regularly performed every 1 to 3 days instead of conventional computed tomography (CT) scans after admission owing to the risk during the transfer of severe patients. A conventional semi-quantitative scoring system for DR was used to evaluate the pulmonary involvement area of the abnormalities including air-space and reticular opacities 19, 20. Each lung was divided into three equidistant zones (upper, middle, and lower), resulting in a total of six zones to be scored. Each zone was visually scored from 0 to 4 as: score 0, 0% involvement; score 1, 1%-25% involvement; score 2, 26%-50% involvement; score 3, 51-75% involvement; and score 4, ≥76% involvement. The DR score was the sum of the score of each zone. The analysis was performed by two radiologists (CZ and LY, who had 26 and 22 years of experience in thoracic radiology, respectively) and the decisions were reached in consensus. All radiologists were blinded to the clinical progress of the patients to avoid information bias.

### Groups and Study Goals

Two groups were divided based on the clinical outcomes: discharge group and death event group. Three objectives were explored in this study: 1. Comparisons of basic characteristics between two groups, including the occurrence of ARDS, which was defined as worsening respiratory symptoms with PaO2/FiO2≤300 mmHg based on the Berlin definition and WHO criteria 21, 22; 2. Risk factors for death on admission; 3. Dynamic changes in DR scores and laboratory investigations between two groups in the follow-up.

### Statistical Analysis

Statistical analysis was performed using IBM SPSS Statistics Software (version 24; IBM, New York, USA). Quantitative data were presented as median with inter-quartile range (IQR) and counting data were presented as the percentage of the total unless otherwise specified. The comparisons of the quantitative data were statistically evaluated using the Mann-Whitney U test, according to the non-normal distribution assessed by the Shapiro-Wilk test. The quantitative parameters were stratified into binary variables with the cutoffs of median values. The comparisons of counting data were evaluated using the Chi-square test. The bivariate and multivariate logistic regressions were used to investigate the risk factors for death events involving the stratified clinical, radiographic, and laboratory parameters with significant differences between two groups on admission, and the odds ratio (OR) with 95% confidence interval (95%CI) were calculated. Variables were excluded in the multivariate logistic regression if: 1. The univariate analysis demonstrated no statistical significance (p<0.05); 2. The multivariate analysis demonstrated the probability of p≥0.10 when using forward-LR method; 3. Variable had obvious colinearity with other variables resulting in variance inflation factor (VIF) of more than 5 23. C-index of predicted probability calculated using the optimal multivariate logistic model was evaluated by ROC curve estimation. A p-value of <0.05 was defined as the statistical significance.

## Results

### Basic characteristics and comparisons between two groups

**Figure 1:**
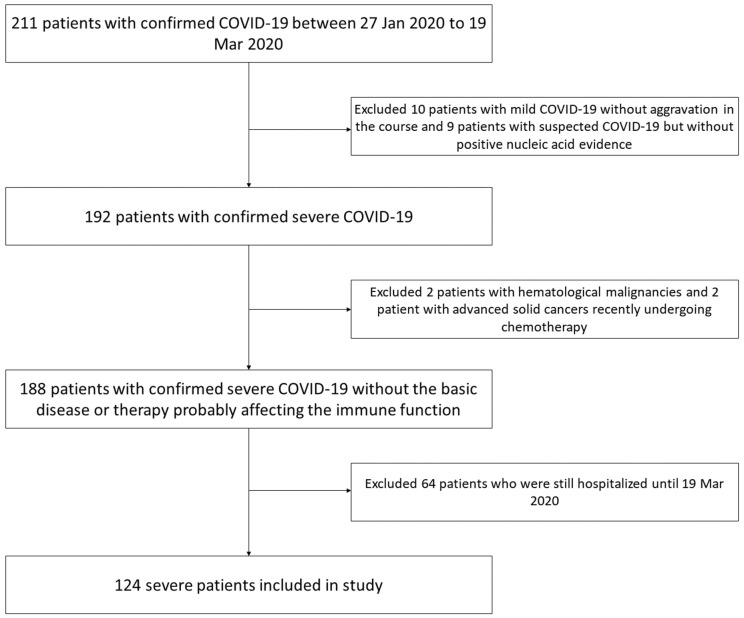
Flowchart of inclusion of the patients.

After retrieving the electronic records, 124 patients were eventually included in the further analysis from 211 consecutive records (**Figure 1**). The basic information of included patients was summarized in **Table 1**. The median age of patients was 68 yrs (IQR 61-75 yrs), and 68.5% of patients were male. Half of the patients had hypertension history and 11 patients had COPD (GOLD II grade) history which was controlled well after quitting smoking and ipratropium treatment. Fever and cough were the most common initial symptoms (85.5% and 69.4%, respectively). The median interval between the admission and symptom onset was 11 days (IQR 7-15 days). On admission, the median body temperature on admission reached 38.3 °C (IQR: 37.0-39.0°C). The median breath rate was 30/min (IQR: 23-33/min) and median SpO2 was 89% (IQR: 82-92%). In most patients (123/124, 99.2%), DRs demonstrated bilateral opacities with the median DR score of 13 (IQR: 7-16). Multiple abnormities of biochemical and hematological parameters were observed as well, including neutrophil, lymphocyte, C-reactive protein (CRP), procalcitonin (PCT), lactate dehydrogenase (LDH), D-dimer, cardiac troponin I (cTnI), and brain natriuretic peptide (BNP), and so on. In the course, 91 patients (73.4%) aggravated to ARDS with the median period of 7 days after admission (IQR 4-11 days), who were transferred to ICU afterwards. Eventually, 89 patients (71.8%) with ARDS complications died who were included in death event group, while 35 patients (28.2%) were recovered and discharged who were included in discharge group.

Compared between the two groups, the patients in death event group had significantly higher male proportion (75.3% vs. 51.4%), lower SpO2 (85% vs. 90%), and higher breath rate (30 vs 25/min) (**Table 2**). In the course, ARDS was more predominantly occurred in death event group compared with discharge group (89 vs. 2). 10 patients with ARDS in death event group refused endotracheal intubation. The median survival period in death event group was 15 days (IQR 9-22 days), while the median hospitalized period in discharge group was 29 days (IQR 23-35 days) (**Table 2**). On admission, the bedside DRs showed larger pulmonary opacities in death event group compared with discharge group, but without a significant difference (14 vs. 11, p=0.289) (**Table 2**). Besides, more abnormities of biochemical and hematological parameters were observed in death event group compared with discharge group, such as neutrophil, lymphocyte, CRP, PCT, LDH, D-dimer, cTnI, and BNP (**Table 2**).

### Risk factor estimation of death events

**Figure 2:**
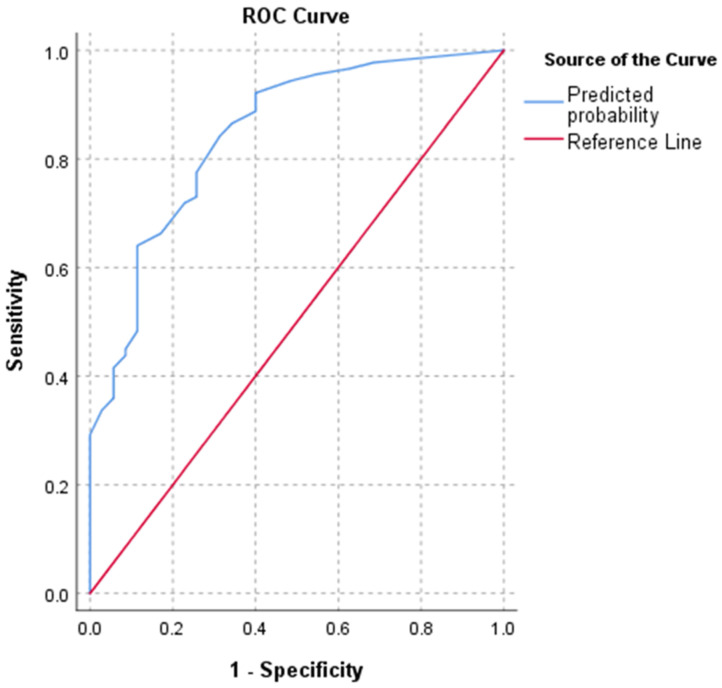
** ROC curve of multivariate logistic regression. Note:** C-index of the predicted probability was 0.845 (p<0.001).

The bivariate logistic regression was performed on the clinical, radiographic, and laboratory parameters (**Table 3**). As a result, ten stratified parameters including male sex, SpO2≤89%, breath rate>30/min, diastolic pressure≤80mmHg, neutrophil>6.46×10^9^/L, lymphocyte≤0.64×10^9^/L, CRP>77.35mg/L, PCT>0.20μg/L, LDH>481U/L, and D-dimer>3.06mg/L showed significant risks with death events (**Table 3**). No obvious multicollinearity was observed with the variance inflation factor (VIF) value of less than 1.6 in each involved variable (**Table S1**). Further multivariate logistic regression showed a significant model fitting involving stratified SpO2, lymphocyte, CRP, PCT, and LDH. The results demonstrated that SpO2≤89%, lymphocyte≤0.64×10^9^/L, CRP>77.35mg/L, PCT>0.20μg/L, and LDH>481U/L were the independent risk factors with the ORs of 2.959, 4.015, 2.852, 3.554, and 3.185, respectively (p<0.04). The C-index of the predicted probability calculated using this multivariate logistic model was 0.845 (p<0.001) (**Figure 2**).

### Dynamic changes in laboratory investigations and DR scores between patients with clinical stability and death events

**Figure 3:**
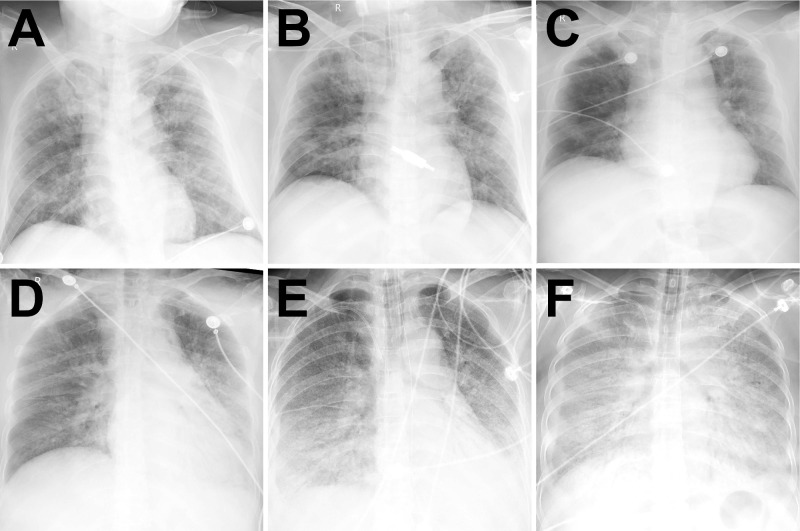
** Typical radiographic aggravation manifestations. Note: A-C** from a patient in discharge group with fever and cough for six days. After admission, chest DR showed a large area of air-space opacity in the bilateral lungs (DR score: 17 points) (**A**). Day 6, worsening respiratory failure (hypoxemia and tachypnea) necessitated intubation and ventilation (Critical aggravation). Chest DR demonstrated progressive diffuse opacity of bilateral lungs (DR score: 17 points) (**B**). Day 11, the intubation was removed and face-mask oxygen treatment was performed. Day 22, chest DR demonstrated partial absorption of the lesions with the reduced density in bilateral lungs (DR score: 14 points) (**C**). The patient eventually recovered and discharged on Day 38. **D-F** from a patient in death event group with fever for one week. After admission, chest DR showed a large area of air-space opacity in the left mid-zone and bilaterally lower zones (DR score: 10 points) (**D**). Day 4, worsening respiratory failure (hypoxemia and tachypnea) necessitated intubation and ventilation (Critical aggravation). Chest DR demonstrated progressive diffuse opacity of bilateral lungs, with bilateral air bronchograms (DR score: 15 points) (**E**). Day 9, chest DR demonstrated complete opacity of bilateral lungs, with bilateral air bronchograms and a right pleural effusion (DR score: 24 points) (**F**). The patient eventually died on Day 12 due to refractory respiratory failure.

**Figure 4:**
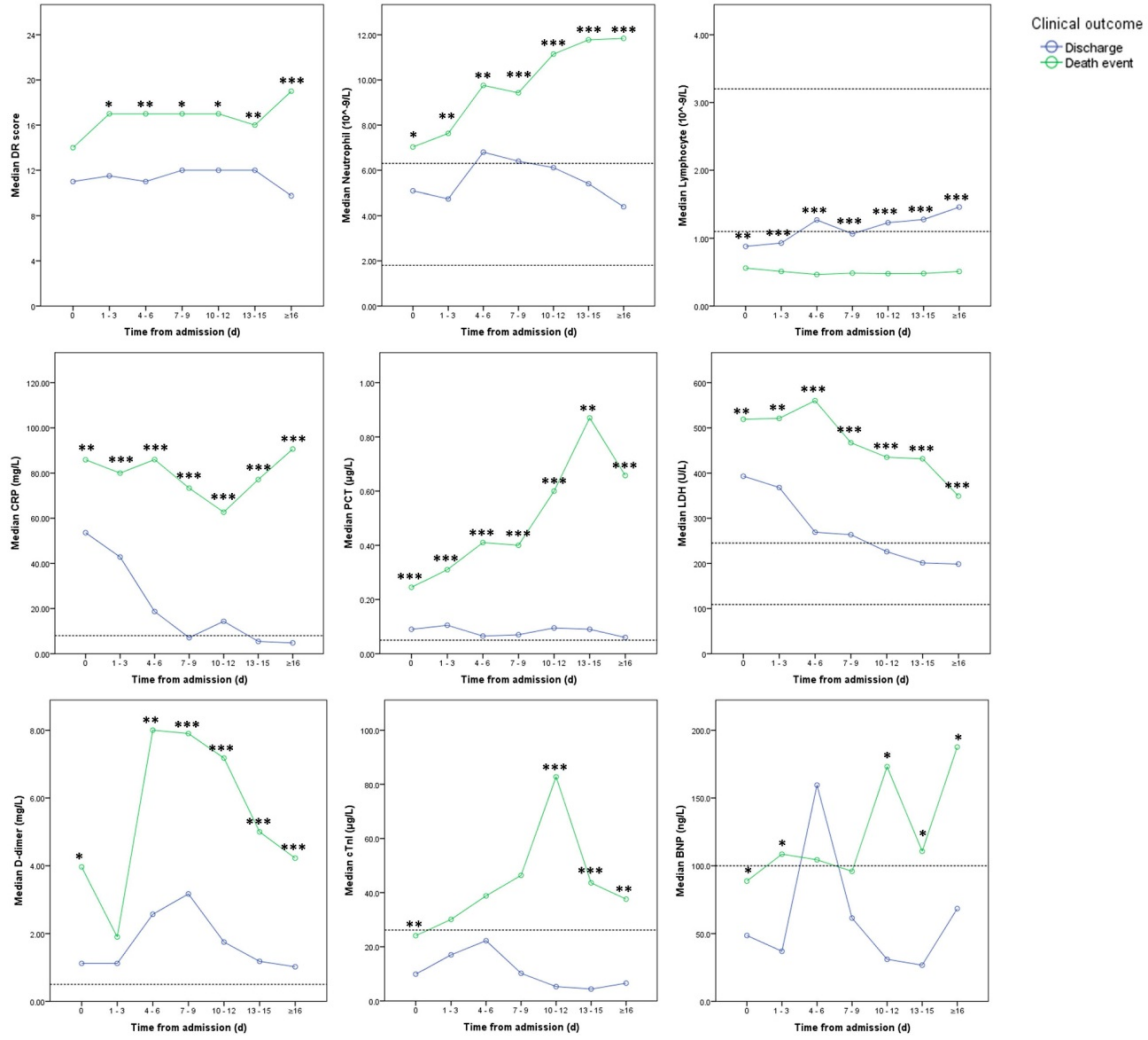
** Dynamic changes in DR scores and laboratory parameters between two groups. Note:** Imaginary lines indicated the normal reference boundary; Man-Whitney U test (*p<0.05; **p<0.01; ***p<0.001).

In the course, DR scores increased in death event group with a significant difference between the two groups after 1 to 3 days from admission (**Figure 3** and** 4**). After 15 days, DR scores slowly declined in discharge group while increasing in death event group (**Figure 3** and **4**). In death event group, the neutrophil gradually increased to more than 10.00×10^9^/L, and lymphocyte stayed at the lower level from 0.47 to 0.56×10^9^/L in the course, with significant differences from discharge group (**Figure 4**). In discharge group, lymphocyte gradually increased from 0.88×10^9^/L to normal level (≥1.1 ×10^9^/L) after nine days. Besides, it demonstrated the significantly higher levels of CRP, PCT, LDH, and D-dimer in death event group, which gradually decreased or stayed at the relatively lower levels in discharge group (**Figure 4**). cTnI and BNP demonstrated dramatic fluctuation with significantly higher levels at some time points in death event group compared with discharge group (**Figure 4**). No obvious abnormalities of total bilirubin (TB), aspartate aminotransferase (AST), and serum creatinine (Scr) in the course were observed in both groups (**Table S2**).

**Figure 5:**
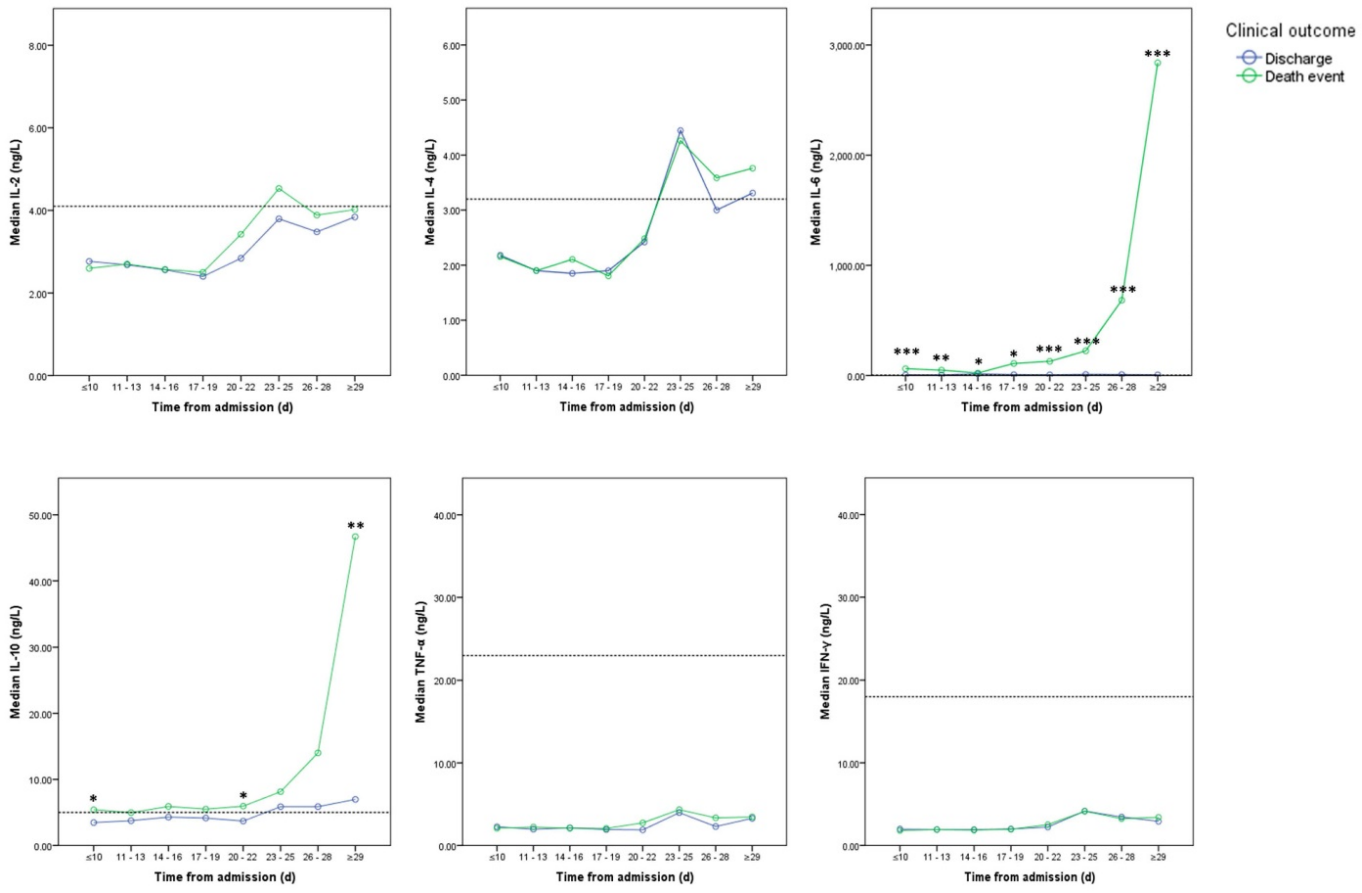
** Dynamic changes in cytokine profile between two groups. Note:** Imaginary lines indicated the normal reference boundary; Man-Whitney U test (*p<0.05; **p<0.01; ***p<0.001).

**Figure 6:**
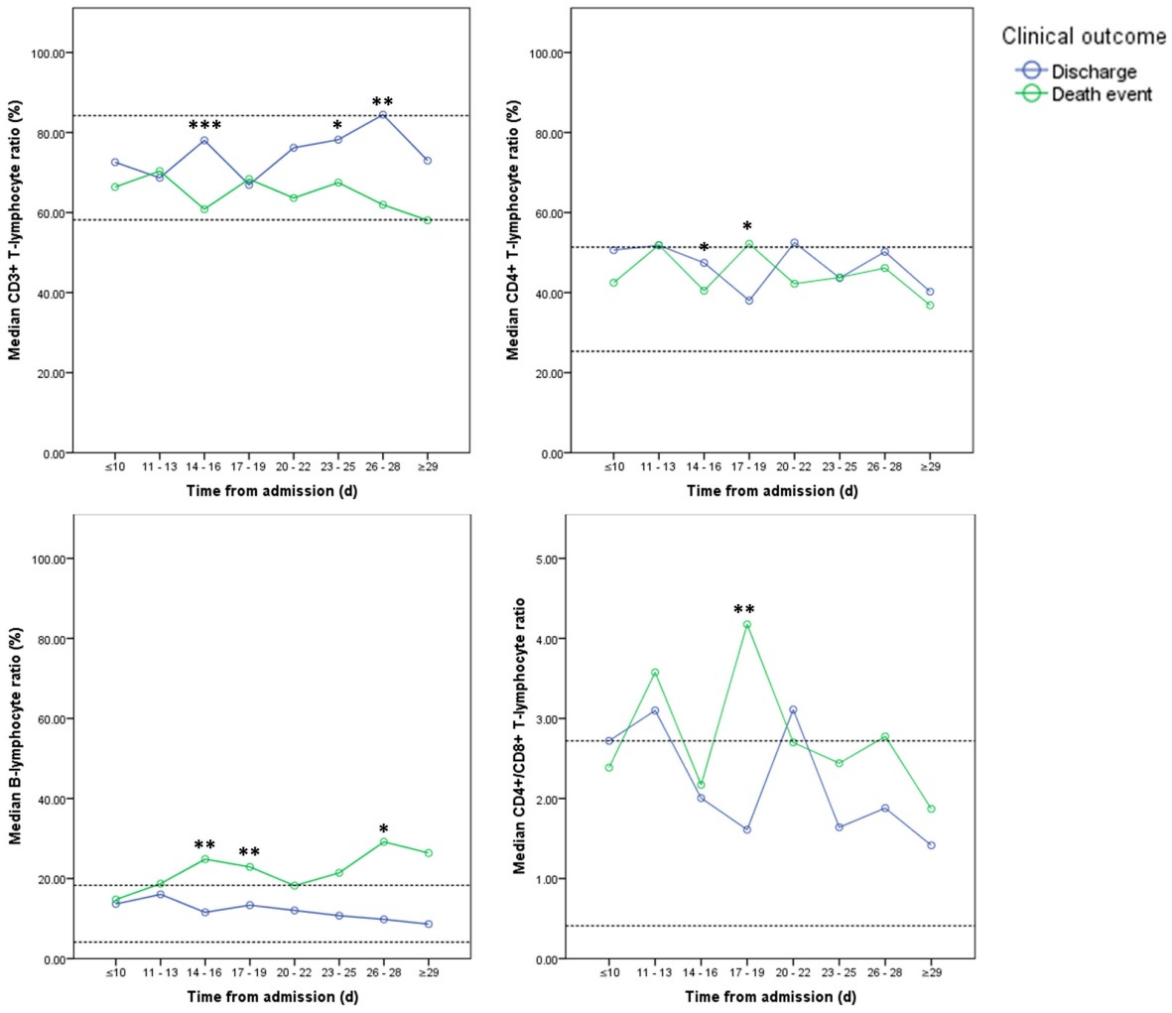
** Dynamic changes in Lymphocyte subsets between two groups. Note:** Imaginary lines indicated the normal reference boundary; Man-Whitney U test (*p<0.05; **p<0.01; ***p<0.001).

Among all 124 patients, 77 patients underwent consecutive cytokine profile and lymphocyte subsets analysis in the course, including 29 patients in charge group and 48 patients in death event group. The levels of IL-2, IL-6, TNF-α, and INF-γ didn't show significant differences between two groups and stayed in the normal reference ranges at most of time (**Figure 5**). Compared with discharge group, IL-6 was significantly elevated in death event group. And IL-10 was significantly increased after 29 days in death event group (**Figure 5**). CD3+, CD4+, and CD8+ T-lymphocyte ratio stayed in the normal range in both groups, though approaching the upper or lower reference at some time points (**Figure 6**). CD3+ and CD8+ T-lymphocyte ratios were significantly higher in discharge group at several time points, but the CD4+/CD8+ T-lymphocyte ratio seemed higher in death group compared with discharge group reaching significant difference at the time point of 17-19 days (**Figure 6**). The quantitative data were summarized in **Table S3**.

## Discussion

This study reported the differences between the discharged and died patients with severe COVID-19 in the clinical course. On admission, SpO2≤89%, lymphocyte≤0.64×10^9^/L, CRP>77.35mg/L, PCT>0.20μg/L, and LDH>481U/L were the independent risk factors associated with death events. Besides, in the clinical course, significantly higher levels of DR score, neutrophil, IL-6, D-dimer, cTnI, and BNP were also observed in death event group. Thus, the results indicated that severe systematic inflammation with cardiac impairment played an important role in the fatal process of COVID-19.

Corresponding to the previous reports, old patients with a median age of 68 yrs (IQR 61-75 yrs) presenting more coexisting illnesses such as hypertension and CVD were more likely to develop severe COVID-19 6, 8. Male patients in this study were more inclined to occurred ARDS and death events, in agreement with a retrospective report with a cohort of 44,672 patients where male patients over 60 years of age with comorbidities had a higher death risk 1. In the course, patients in death event group showed a significantly higher ratio of ARDS than discharge group (100.0% vs. 5.7%). It indicated the survival rate was very low if the patients aggravated to this critical status and further exploration of the treatment to prevent ARDS is imperative. Although no significant difference in the period between initial symptom onset and admission was found between two groups, attention still should be paid to early diagnosis and treatment because the mean period of 11 days from symptom onset to admission in this study was probably too late when the severe progression had occurred 9.

Because the DR demonstrated a good consistency to chest CT which was also capable of estimating the COVID-19 in the follow-up, it was consecutively performed in this cohort in order to reduce the transferring risk of the severe patients and X-ray exposure 24. On admission, bilaterally diffuse pulmonary opacities in DRs were observed in most patients (99.5%), similar to the demonstrations in SARS and MERS 19, 20. The higher DR scores in death event group on admission were presented with a lower SpO2 level and a higher breath rate when compared with discharge group, though no significant difference was revealed. However, more rapid progression of pulmonary opacities with a significantly higher DR score was observed afterwards in death event group. Different from no absorption in death event group, the pulmonary lesions were slowly absorbed after half a month from admission in discharge group. Due to the limited medical resources in this provisional center, lung ultrasound was not routinely performed. From the previous studies, lung ultrasound could also be performed at the bedside and has been used in diagnosing and monitoring pneumonia with higher sensitivity and specificity (≥94%) than DR but without X-ray exposure 25, 26. Thus, it might be a good alternative to conventional bedside DR in severe COVID-19 patients.

Up to date, some previous studies already revealed multiple risk factors associated with poor prognosis, such as compromised respiratory status, older age, male sex, lymphocytopenia, high Sequential Organ Failure Assessment score, and elevated levels of CRP, PCT, LDH, and D-dimer 1, 5-8, 10-12. However, these studies did not consider the impacts of the disease severities on admission in the analysis. As a complementary, this study provided a risk-factor analysis only in the population of severe COVID-19 who presented a higher mortality rate than moderate COVID-19 8. As a case-control study, the bivariate logistic regression returned with ten stratified risk factors for death with statistical significances, including male sex, SpO2≤89%, breath rate>30/min, diastolic pressure≤80mmHg, neutrophil>6.46×10^9^/L, lymphocyte≤0.64×10^9^/L, CRP>77.35mg/L, PCT>0.20μg/L, LDH>481U/L, and D-dimer>3.06mg/L. Further multivariate logistic regression using the forward-LR method resulted in a significant fitting model with good discriminatory power (C-index=0.845). The results revealed SpO2≤89%, lymphocyte≤0.64×10^9^/L, CRP>77.35mg/L, PCT>0.20μg/L, and LDH>481U/L on admission were the independent risk factors, among which the lymphocyte≤0.64×10^9^/L demonstrated the highest OR of 4.015. Moreover, the abnormalities of lymphocyte, CRP, PCT, and LDH stayed or continuously aggravated in death event group but gradually recovered to normal levels in discharge group. Therefore, the dynamic monitoring of these laboratory parameters could estimate the prognosis in clinical practice for severe patients.

In further analysis of the lymphocyte subsets in limited groups, no obvious abnormalities of CD3+, CD4+, and CD8+ T-lymphocyte ratio were found in both groups. However, relatively high CD4+/CD8+ T-lymphocyte ratio above or near the upper normal reference was observed in both groups, while it seemed higher in death event group. Therefore, it indicated no obvious impairment of the immune function, though lymphocyte reduced in severe COVID-19. As an evidence, a previous post-mortem autopsy study of COVID-19, it revealed reduced CD4+ and CD8+ T-lymphocyte in peripheral blood with overactivation of T-lymphocyte manifested by an increase of Th17 and high cytotoxicity of CD8+ T-lymphocytes 5. As a result, severe immune injury could occur in severe patients.

To date, no confirmed evidence of the viral attack on solid organs other than lung was observed in pathological analysis and autopsy 5, 27. Two previous studies in the same center found significantly elevated levels of multiple serum cytokines in severe COVID-19 patients, such as IL-1β, IL-2, IL-6, TNF-α, and INF-γ, which were associated with the disease severity 3, 7. It was suspected a phenomenon named “cytokine storm” might result in the progression of the disease in viral pneumonia 28, 29. In previous studies, COVID-19 was thought to induce “cytokine storm” that then caused multi-organ impairment involving the heart, liver, and kidney 6, 7, 10, 13. However, the clinical course of our patients did not appear to follow this sequence of events. Among multiple cytokines, only IL-6 as one major proinflammatory factor significantly increased with time in death event group with the simultaneous increase of IL-10 as an anti-inflammatory factor, while IL-2, IL-4, TNF-α, and INF-γ neither showed significant differences between two groups nor demonstrated obvious abnormalities in the course. Besides, the hepatic and renal related parameters were not significantly elevated. Therefore, the result of this study didn't support this fatal hypothesis of “cytokine storm” with induced multi-organ impairments in severe COVID-19. It was speculated that in those published studies, the dynamic changes of the related laboratory parameters were demonstrated as the mean value which might be not appropriate due to the non-normal distribution of the data 6, 10. Thus, the dynamic demonstration with the median value was more reliable in this study.

On the other hand, CRP, PCT, IL-6, and BNP as the major indicators of inflammatory severity in intensive care medicine, had significantly increased or stayed at a relatively high level in death event group, corresponding to the aggravated sepsis in these patients which could cause life-threatening organ dysfunction 30, 31. As indirect evidence, increased CD4+/CD8+ T-lymphocyte ratio in death group was also a typical abnormality in sepsis 32. Meanwhile, gradually increased neutrophil was observed indicating progressive inflammation as part of the inadequate host response to viral aggression 33. In summary, all of these abnormalities could be ascribed to severe systematic inflammation caused by COVID-19. More than this, the virus-induced inflammation could initiate the activation of the fibrinolytic system and fibrin catabolism resulting in the elevation of the D-dimer level in severe patients which was also observed in this study 6, 11, 34. As a complication, a persistently high level of LDH, cTnI, and BNP indicated potential cardiac impairment in the course like reported in other studies 7, 13. However, the elevated levels of D-dimer, cTnI, and BNP fluctuated or decreased in the course but not persistently aggravated, while these parameters progressively deteriorated with time in non-survivors in previous studies 6, 11. As speculation, these differences might be ascribed to more attention paid to the cardiac protection and microcirculation improvement for the patients in this cohort.

In brief, it was concluded that the systematic inflammation with induced cardiac dysfunction was the leading cause of death events in severe COVID-19 except for ARDS. Thus, the use of corticosteroids should still be considered for critical patients to prevent severe inflammatory response 15-17. In one recent study, it was found treatment with methylprednisolone significantly decreased death risk 12. Serum cytokine profiling and lymphocyte subsets analysis might be useful to guide the immunomodulatory treatment 35.

This case-controlled study has limitations. First, the sample size is limited in this study and not all patients underwent sequential cytokine profile and lymphocyte subsets tests. Second, the study was limited by the heterogeneity of treatments based on the disease status with time, which was impossible to summarize in this cohort. Thus, only the speculation was made to explain the fluctuation of some abnormal laboratory parameters in the course. Third, the dynamic changes of vital signs and oxygen saturation were not presented, because they were continuously monitored in the course and sort of affected by the adjusted treatments. At last, some important subsets of lymphocytes including Th17 and Treg were not measured due to the limitation of the laboratory techniques in this provisional isolation center, thus the identification of the immune function was not comprehensive.

## Conclusions

In summary, this study revealed the clinical course of severe COVID-19 patients from admission with different outcomes. Dynamic monitoring of lymphocyte, CRP, PCT, and LDH could be meaningful to predict the prognosis of severe patients. Besides, the fatal feature of COVID-19 is mostly attributed to severe systematic inflammation with induced cardiac dysfunction except for ARDS.

## Supplementary Material

Supplementary figures and tables.

## Figures and Tables

**Table 1:** Baseline characteristics.

	Normal reference range	Total (n=124)
**Age (y)**		68 (61-75)
**Sex**		
Male		85 (68.5)
Female		39 (31.5)
**Medical history**		
Hypertension		62 (50.0)
Cardiovascular disease (CVD)		19 (15.3)
Diabetes mellitus		25 (20.2)
Chronic obstructive pulmonary disease (COPD)		11 (8.9)
**Initial symptoms**		
Fever		106 (85.5)
Low grade fever (37.5 - 38.0 ℃)		24 (19.4)
Moderate fever (38.1 - 39.0 ℃)		42 (33.9)
High-grade fever (≥39.1 ℃)		40 (32.3)
Cough		86 (69.4)
Fatigue		61 (49.2)
Chest distress		57 (46.0)
Expectoration		49 (39.5)
Chills		27 (21.8)
Myalgia		26 (21.0)
Diarrhea		19 (15.3)
**Time of admission after symptom onset (d)**		11 (7-15)
**Vital signs on admission**		
SpO2 (%)		89 (82-92)
Body temperature (℃)		38.3 (37.0-39.0)
Heart rate (/min)		90 (80-102)
Breath rate (/min)		30 (23-33)
Systolic pressure (mmHg)		132 (122-150)
Diastolic pressure (mmHg)		80 (73-88)
**Diagnosis of severe COVID-19**		
Breath rate≥30/min		6 (4.8)
SpO2≤93%		58 (46.8)
Both		60 (48.4)
**DR estimations on admission**		
Unilateral involvement		1 (0.8)
Bilateral involvement		123 (99.2)
DR score	(0)	13 (7-16)
**Laboratory investigations on admission**		
White blood cell (WBC) (×10^9^/L)	(3.50-9.50)	7.52 (5.07-10.92)
Neutrophil (×10^9^/L)	(1.80-6.30)	6.46 (3.91-9.92)
Lymphocyte (×10^9^/L)	(1.10-3.20)	0.64 (0.46-1.02)
Hemoglobin (g/L)	(115-150)	130 (121-142)
Platelet (×10^9^/L)	(125-350)	187 (150-230)
C-reactive protein (CRP) (mg/L)	(0.00-8.00)	77.35 (43.13-111.94)
Procalcitonin (PCT) (μg/L)	(<0.05)	0.20 (0.09-0.40)
Total bilirubin (TB) (μmol/L)	(3.0-20.0)	13.6 (9.7-20.0)
Alanine aminotransferase (ALT) (U/L)	(5-35)	37 (25-59)
Aspartate aminotransferase (AST) (U/L)	(8-40)	47 (31-69)
Lactate dehydrogenase (LDH) (U/L)	(109-245)	481 (360-597)
Albumin (g/L)	(33.0-55.0)	28.2 (26.0-30.9)
Globulin (g/L)	(20.0-35.0)	34.2 (31.0-38.0)
Serum creatinine (Scr) (μmol/L)	(41.0-81.0)	74.1 (62.2-99.7)
Na+ (mmol/L)	(137.0-147.0)	138.3 (135.8-140.4)
K+ (mmol/L)	(3.5-5.3)	3.87 (3.53-4.24)
Cl- (mmol/L)	(96-108)	102.3 (99.6-105.2)
D-dimer (mg/L)	(<0.5)	3.06 (0.62-8.00)
Activated partial thromboplastin time (APTT) (s)	(27.0-45.0)	38.0 (33.1-42.5)
Prothrombin time (PT) (s)	(11.0-16.0)	13.9 (13.0-14.9)
Fibrinogen (g/L)	(2.00-4.00)	4.58 (3.45-5.25)
Cardiac troponin I (cTnI) (μg/L)	(<26.2)	19.3 (8.4-96.4)
Brain natriuretic peptide (BNP) (ng/L)	(<100.0)	79.3 (30.4-164.5)
**Occurrence of ARDS in the course**		91 (73.4)
**Period between admission and occurrence of ARDS (d)**		7 (4-11)
**Hospitalized period (d)**		19 (11-27)

**Abbreviations:** CVD: cardiovascular disease; COPD: chronic obstructive pulmonary disease; DR: digital radiograph; WBC: white blood cell count; CRP: C-reactive protein; PCT: procalcitonin; TB: total bilirubin; ALT: alanine aminotransferase; AST: aspartate aminotransferase; LDH: lactate dehydrogenase; Scr: serum creatinine; APTT: activated partial thromboplastin time; PT: prothrombin time; cTnI: cardiac troponin I; BNP: brain natriuretic peptide.

**Table 2:** Comparisons between two groups Initial radiological estimations and laboratory investigations on admission.

	Discharge group (n=35)	Death events group (n=89)	*p*-value
**Age (y)**	65 (49-77)	69 (61-73)	0.285
≤68	20 (57.1)	42 (47.2)	0.318
>68	15 (42.9)	47 (52.8)	
**Sex**			
Male	18 (51.4)	67 (75.3)	**0.010**
Female	17 (48.6)	22 (24.7)	
**Medical history**			
Hypertension	15 (42.9)	47 (52.8)	0.318
CVD	6 (17.1)	13 (14.6)	0.724
Diabetes mellitus	6 (17.1)	19 (21.3)	0.599
COPD	4 (11.4)	7 (7.9)	0.530
**Initial symptoms**			
Fever	31 (88.6)	75 (84.3)	0.540
* Low grade fever (37.5 - 38.0 ℃)*	10 (28.6)	14 (15.7)	0.432
* Moderate fever (38.1 - 39.0 ℃)*	11 (31.4)	31 (34.8)	
* High-grade fever (≥39.1 ℃)*	10 (28.6)	30 (33.7)	
Cough	28 (80.0)	58 (65.2)	0.107
Fatigue	16 (45.7)	45 (50.6)	0.627
Chest distress	15 (42.9)	42 (47.2)	0.663
Expectoration	14 (40.0)	35 (39.3)	0.945
Chills	6 (17.1)	21 (23.6)	0.433
Myalgia	6 (17.1)	20 (22.5)	0.512
Diarrhea	2 (5.7)	17 (19.1)	0.063
**Time of admission after symptom onset (d)**	11 (9-15)	10 (7-15)	0.354
≤11	21 (60.0)	48 (53.9)	0.540
>11	14 (40.0)	41 (46.1)	
**Vital signs after admission**			
SpO2 (%)	90 (88-92)	85 (80-90)	**0.001**
* ≤89*	9 (25.7)	53 (59.6)	**0.001**
* >89*	26 (74.3)	36 (40.4)	
Body temperature (℃)	38.0 (36.6-39.0)	38.4 (37.4-39.0)	0.237
* ≤38.3*	19 (54.3)	44 (49.4)	0.627
* >38.3*	16 (45.7)	45 (50.6)	
Heart rate (/min)	87 (76-103)	92 (80-102)	0.467
* ≤90*	21 (60.0)	42 (47.2)	0.199
* >90*	14 (40.0)	47 (52.8)	
Breath rate (/min)	25 (20-30)	30 (25-34)	**0.001**
* ≤30*	30 (85.7)	54 (60.7)	**0.007**
* >30*	5 (14.3)	35 (39.3)	
Systolic pressure (mmHg)	131 (121-145)	132 (122-150)	0.683
* ≤132*	20 (57.1)	46 (51.7)	0.584
* >132*	15 (42.9)	43 (48.3)	
Diastolic pressure (mmHg)	85 (75-90)	79 (73-87)	0.090
* ≤80*	14 (40.0)	54 (60.7)	**0.037**
* >80*	21 (60.0)	35 (39.3)	
**Occurrence of ARDS in the course**	2 (5.7)	89 (100.0)	**<0.001**
**Treatments**			
Nasal/mask oxygen therapy	33 (94.3)	10 (11.2)	**<0.001**
Mechanical ventilation	2 (5.7)	79 (88.8)	
**Hospitalized period (d)**	29 (23-35)	15 (9-22)	**<0.001**
**Radiological estimations**			
Unilateral involvement	1 (2.9)	0 (0)	0.282
Bilateral involvement	34 (97.1)	89 (100)	
DR score	11 (8-15)	14 (7-17)	0.398
* ≤13*	21 (60.0)	44 (49.4)	0.289
* >13*	14 (40.0)	45 (50.6)	
**Laboratory investigations**			
WBC (×10^9^/L)	6.55 (4.94-9.43)	8.09 (5.20-11.11)	0.086
* ≤7.52*	22 (62.9)	40 (44.9)	0.073
* >7.52*	13 (37.1)	49 (55.1)	
Neutrophil (×10^9^/L)	5.09 (3.73-8.26)	7.03 (4.17-10.23)	**0.037**
* ≤6.46*	25 (71.4)	37 (41.6)	**0.003**
* >6.46*	10 (28.6)	52 (58.4)	
Lymphocyte (×10^9^/L)	0.88 (0.66-1.21)	0.56 (0.39-0.83)	**0.001**
* ≤0.64*	8 (22.9)	55 (61.8)	**<0.001**
* >0.64*	27 (77.1)	34 (38.2)	
Hemoglobin (g/L)	126 (120-137)	131 (122-144)	0.066
* ≤130*	22 (62.9)	43 (48.3)	0.144
* >130*	13 (37.1)	46 (51.7)	
Platelet (×10^9^/L)	197 (143-234)	182 (151-229)	0.407
* ≤187*	15 (42.9)	48 (53.9)	0.267
* >187*	20 (57.1)	41 (46.1)	
CRP (mg/L)	53.57 (30.28-78.56)	85.86 (51.35-116.83)	**0.001**
* ≤77.35*	26 (74.3)	37 (41.6)	**0.001**
* >77.35*	9 (25.7)	52 (58.4)	
PCT (μg/L)	0.09 (0.06-0.17)	0.25 (0.12-0.44)	**<0.001**
* ≤0.20*	26 (74.3)	36 (40.4)	**0.001**
* >0.20*	9 (25.7)	53 (59.6)	
TB (μmol/L)	12.1 (7.9-16.4)	14.1 (9.9-21.3)	0.082
* ≤13.6*	19 (54.3)	43 (48.3)	0.549
* >13.6*	16 (45.7)	46 (51.7)	
ALT (U/L)	37 (22-78)	37 (25-56)	0.936
* ≤37*	18 (51.4)	45 (50.6)	0.931
* >37*	17 (48.6)	44 (49.4)	
AST (U/L)	44 (27-73)	47 (36-67)	0.361
* ≤47*	19 (54.3)	45 (50.6)	0.709
* >47*	16 (45.7)	44 (49.4)	
LDH (U/L)	393 (244-497)	519 (395-634)	**0.001**
* ≤481*	26 (74.3)	36 (40.4)	**0.001**
* >481*	9 (25.7)	53 (59.6)	
Albumin (g/L)	28.8 (26.6-32.0)	28.0 (25.7-30.6)	0.165
* ≤28.2*	15 (42.9)	48 (53.9)	0.267
* >28.2*	20 (57.1)	41 (46.1)	
Globulin (g/L)	34.7 (30.6-37.8)	33.9 (31.0-38.3)	0.936
* ≤34.2*	17 (48.6)	45 (50.6)	0.842
* >34.2*	18 (51.4)	44 (49.4)	
Scr (μmol/L)	70.0 (54.4-88.2)	75.5 (64.3-102.5)	0.147
* ≤74.1*	20 (57.1)	42 (47.2)	0.318
* >74.1*	15 (42.9)	47 (52.8)	
D-dimer (mg/L)	1.12 (0.43-5.42)	3.97 (0.78-8.00)	**0.010**
* ≤3.06*	24 (68.6)	39 (43.8)	**0.013**
* >3.06*	11 (31.4)	50 (56.2)	
APTT (s)	38.5 (34.4-43.1)	37.4 (32.7-42.3)	0.286
* ≤38.0*	16 (45.7)	46 (51.7)	0.549
* >38.0*	19 (54.3)	43 (48.3)	
PT (s)	13.7 (12.9-14.4)	14.2 (13.0-15.2)	0.147
* ≤13.9*	21 (60.0)	41 (46.1)	0.163
* >13.9*	14 (40.0)	48 (53.9)	
Fibrinogen (g/L)	4.68 (3.75-5.19)	4.58 (3.23-5.25)	0.784
* ≤4.58*	17 (48.6)	45 (50.6)	0.842
* >4.58*	18 (51.4)	44 (49.4)	
cTnI (μg/L)	9.9 (3.4-57.1)	24.1 (9.8-155.8)	**0.006**
* ≤19.3*	20 (57.1)	41 (46.1)	0.267
* >19.3*	15 (42.9)	48 (53.9)	
BNP (ng/L)	48.6 (21.1-119.5)	88.7 (39.6-192.0)	**0.029**
* ≤79.3*	21 (60.0)	41 (46.1)	0.163
* >79.3*	14 (40.0)	48 (53.9)	

**Abbreviations:** CVD: cardiovascular disease; COPD: chronic obstructive pulmonary disease; DR: digital radiograph; WBC: white blood cell count; CRP: C-reactive protein; PCT: procalcitonin; TB: total bilirubin; ALT: alanine aminotransferase; AST: aspartate aminotransferase; LDH: lactate dehydrogenase; Scr: serum creatinine; APTT: activated partial thromboplastin time; PT: prothrombin time; cTnI: cardiac troponin I; BNP: brain natriuretic peptide.

**Table 3:** Bivariate and multivariate logistic regression of risk factors associated with death events.

	Bivariate		Multivariate	
	OR (95% CI)	p value	OR (95% CI)	p value
**Age (y) (>68 vs. ≤68)**	1.492 (0.678-3.282)	0.320		
**Sex (male vs. female)**	2.876 (1.268-6.526)	**0.011**		
**Time of admission after symptom onset (d) (>11 vs. ≤11)**	1.281 (0.579-2.835)	0.541		
**SpO2 (%) (≤89 vs. >89)**	4.253 (1.785-10.133)	**0.001**	2.959 (1.072-8.167)	**0.036**
**Body temperature (℃) (>38.3 vs. ≤38.3)**	1.214 (0.554-2.660)	0.627		
**Heart rate (/min) (>89 vs. ≤89)**	1.679 (0.759-3.714)	0.201		
**Breath rate (/min) (>30 vs. ≤30)**	3.889 (1.377-10.979)	**0.010**		
**Systolic pressure (mmHg) (≤132 vs. >132)**	1.246 (0.567-2.741)	0.584		
**Diastolic pressure (mmHg) (≤80 vs. >80)**	2.314 (1.041-5.145)	**0.040**		
**DR score (>13 vs. ≤13)**	1.534 (0.694-3.393)	0.291		
**WBC (×10^9^/L) (>7.52 vs. ≤7.52)**	2.073 (0.929-4.627)	0.075		
**Neutrophil (×10^9^/L) (>6.46 vs. ≤6.46)**	3.514 (1.508-8.187)	**0.004**		
**Lymphocyte (×10^9^/L) (≤0.64 vs. >0.64)**	5.460 (2.226-13.393)	**<0.001**	4.015 (1.457-11.065)	**0.007**
**Hemoglobin (g/L) (≤130 vs. >130)**	0.552 (0.248-1.232)	0.147		
**Platelet (×10^9^/L) (≤187 vs. >187)**	1.561 (0.709-3.435)	0.268		
**CRP (mg/L) (>77.35 vs. ≤77.35)**	4.060 (1.706-9.665)	**0.002**	2.852 (1.054-7.715)	**0.039**
**PCT (μg/L) (>0.20 vs. ≤0.20)**	4.253 (1.785-10.133)	**0.001**	3.554 (1.284-9.834)	**0.015**
**TB (μmol/L) (>13.6 vs. ≤13.6)**	1.270 (0.580-2.783)	0.550		
**ALT (U/L) (>37 vs. ≤37)**	1.035 (0.473-2.264)	0.931		
**AST (U/L) (>47 vs. ≤47)**	1.161 (0.530-2.544)	0.709		
**LDH (U/L) (>481 vs. ≤481)**	4.253 (1.785-10.133)	**0.001**	3.185 (1.154-8.788)	**0.025**
**Albumin (g/L) (≤28.2 vs. >28.2)**	1.561 (0.709-3.435)	0.268		
**Globulin (g/L) (≤34.2 vs. >34.2)**	1.083 (0.495-2.368)	0.842		
**Scr (μmol/L) (>74.1 vs. ≤74.1)**	1.492 (0.678-3.282)	0.320		
**D-dimer (mg/L) (>3.06 vs. ≤3.06)**	2.797 (1.223-6.398)	**0.015**		
**APTT (s) (>38.0 vs. ≤38.0)**	1.270 (0.580-2.783)	0.550		
**PT (s) (>13.9 vs. ≤13.9)**	1.756 (0.794-3.886)	0.165		
**Fibrinogen (g/L) (≤4.58 vs. >4.58)**	1.083 (0.495-2.368)	0.842		
**cTnI (μg/L) (>19.3 vs. ≤19.3)**	1.561 (0.709-3.435)	0.268		
**BNP (ng/L) (>79.3 vs. ≤79.3)**	1.756 (0.794-3.886)	0.165		

**Abbreviations:** DR: digital radiograph; WBC: white blood cell count; CRP: C-reactive protein; PCT: procalcitonin; TB: total bilirubin; ALT: alanine aminotransferase; AST: aspartate aminotransferase; LDH: lactate dehydrogenase; Scr: serum creatinine; APTT: activated partial thromboplastin time; PT: prothrombin time; cTnI: cardiac troponin I; BNP: brain natriuretic peptide.

## Abbreviations

COVID-19: coronavirus disease-19
SARS: Severe Acute Respiratory Syndrome
MERS: Middle East Respiratory Syndrome
ARDS: acute respiratory distress syndrome
ICU: intensive care unit
DR: digital radiograph
CT: computed tomography
IQR: inter-quartile range
OR: odds ratio
95%CI: 95% confidence interval
VIF: variance inflation factor
CVD: cardiovascular disease
COPD: chronic obstructive pulmonary disease
DR: digital radiograph
WBC: white blood cell count
CRP: C-reactive protein
PCT: procalcitonin
TB: total bilirubin
ALT: alanine aminotransferase
AST: aspartate aminotransferase
LDH: lactate dehydrogenase
Scr: serum creatinine
APTT: activated partial thromboplastin time
PT: prothrombin time
cTnI: cardiac troponin I
BNP: brain natriuretic peptide
