# Supplementary material for: Factors associated with death outcome in patients with severe coronavirus disease-19 (COVID-19): a case-control study
Source: Int J Med Sci. 2020 May 18;17(9):1281–92. doi: 10.7150/ijms.46614 (PMC7294915; doi:10.7150/ijms.46614)
Supplement: Supplementary file 1 — Supplementary figures and tables. [file ijmsv17p1281s1.pdf]

SUPPLEMENTARY MATERIALS

Table S1: Collinearity Statistics in multivariate logistic regression.

	Tolerance	VIF
**Sex**	0.771	1.296
**SpO2**	0.841	1.188
**Breath rate**	0.856	1.168
**Diastolic pressure**	0.685	1.460
**DR score**	0.691	1.446
**Neutrophil**	0.656	1.525
**Lymphocyte**	0.833	1.201
**C-reactive protein (CRP)**	0.767	1.304
**Procalcitonin (PCT)**	0.851	1.174
**Lactate dehydrogenase (LDH)**	0.763	1.311
**D-dimer**	0.750	1.333

Table S2: Dynamic changes in laboratory investigations and DR scores between patients with clinical stability and death events.S

Time from admission (d)	Discharge (n=35)	Death event (n=89)	p value
**DR score**			
0	11 (8-15)	14 (7-17)	0.398
1 - 3	12 (7-15)	17 (16-20)	0.047
4 - 6	11 (6-15)	17 (14-19)	0.004
7 - 9	12 (8-18)	17 (12-20)	0.035
10 - 12	12 (8-19)	17 (12-20)	0.047
13 - 15	12 (7-14)	16 (12-21)	0.009
≥16	10 (6-14)	19 (13-21)	<0.001
**Neutrophil (G/L)**			
0	5.09 (3.73-8.26)	7.03 (4.17-10.23)	0.037
1 - 3	4.74 (3.45-6.40)	7.64 (4.78-12.65)	0.002
4 - 6	6.80 (4.76-8.27)	9.76 (6.18-13.10)	0.002
7 - 9	6.40 (3.73-7.66)	9.43 (6.98-13.16)	<0.001
10 - 12	6.12 (4.17-7.95)	11.15 (7.88-13.70)	<0.001
13 - 15	5.40 (3.25-8.71)	11.78 (7.13-16.73)	<0.001
≥16	4.40 (3.23-7.61)	11.84 (7.93-15.57)	<0.001
**Lymphocyte (G/L)**			
0	0.88 (0.66-1.21)	0.56 (0.39-0.83)	0.001
1 - 3	0.93 (0.70-1.21)	0.51 (0.38-0.73)	<0.001
4 - 6	1.27 (0.68-1.51)	0.47 (0.34-0.71)	<0.001
7 - 9	1.07 (0.74-1.72)	0.49 (0.35-0.72)	<0.001
10 - 12	1.23 (0.69-1.61)	0.48 (0.32-0.75)	<0.001
13 - 15	1.28 (0.79-1.78)	0.48 (0.27-0.86)	<0.001
≥16	1.46 (0.91-1.88)	0.51 (0.25-0.93)	<0.001
**C-reactive protein (mg/L)**			
0	53.57 (30.28-78.56)	85.86 (51.35-116.83)	0.001
1 - 3	42.82 (12.48-63.71)	79.97 (45.67-117.28)	<0.001
4 - 6	18.67 (4.76-54.59)	85.96 (57.93-109.74)	<0.001
7 - 9	7.11 (2.95-23.39)	73.31 (47.01-114.48)	<0.001
10 - 12	14.33 (3.91-51.00)	62.67 (39.32-130.58)	<0.001
13 - 15	5.45 (3.86-20.22)	77.10 (42.87-144.87)	<0.001
≥16	4.79 (0.36-12.36)	90.62 (64.42-114.31)	<0.001
**Procalcitonin (PCT) (μg/L)**			
0	0.09 (0.06-0.17)	0.25 (0.12-0.44)	<0.001
1 - 3	0.11 (0.07-0.23)	0.31 (0.18-0.65)	<0.001
4 - 6	0.07 (0.05-0.14)	0.41 (0.16-1.03)	<0.001
7 - 9	0.07 (0.06-0.12)	0.40 (0.15-0.77)	<0.001
10 - 12	0.10 (0.05-0.12)	0.60 (0.20-2.19)	<0.001
13 - 15	0.09 (0.04-0.15)	0.87 (0.17-2.11)	0.001

≥16	0.06 (0.05-0.10)	0.66 (0.29-1.05)	<0.001
**Total bilirubin (TB) (μmol/L)**			
0	12.1 (7.9-16.4)	14.1 (9.9-21.3)	0.082
1 - 3	10.5 (9.0-17.9)	13.7 (9.7-21.5)	0.129
4 - 6	10.9 (9.1-15.7)	18.2 (12.9-31.8)	<0.001
7 - 9	12.2 (9.0-14.4)	17.7 (12.8-29.5)	0.006
10 - 12	12.4 (8.8-20.1)	17.2 (13.2-25.1)	0.025
13 - 15	12.6 (9.5-15.4)	16.4 (11.2-25.9)	0.007
≥16	10.3 (8.5-13.8)	15.2 (10.1-22.8)	0.009
**Alanine aminotransferase (ALT) (U/L)**			
0	37 (22-78)	37 (25-56)	0.936
1 - 3	39 (27-81)	40 (27-66)	0.780
4 - 6	45 (25-64)	43 (29-93)	0.527
7 - 9	41 (32-55)	42 (29-75)	0.575
10 - 12	43 (28-56)	43 (26-65)	0.875
13 - 15	42 (27-51)	49 (23-85)	0.321
≥16	29 (26-55)	47 (27-79)	0.087
**Aspartate aminotransferase (AST) (U/L)**			
0	44 (27-73)	47 (36-67)	0.361
1 - 3	34 (26-78)	47 (36-70)	0.187
4 - 6	26 (20-41)	42 (26-75)	0.004
7 - 9	29 (21-35)	36 (28-61)	0.009
10 - 12	28 (23-37)	39 (23-66)	0.026
13 - 15	27 (20-32)	38 (27-78)	0.004
≥16	23 (21-30)	34 (24-41)	0.003
**Lactate dehydrogenase (LDH) (U/L)**			
0	393 (244-497)	519 (395-634)	0.001
1 - 3	368 (261-481)	521 (382-650)	0.001
4 - 6	269 (207-414)	560 (392-729)	<0.001
7 - 9	264 (193-309)	467 (363-709)	<0.001
10 - 12	226 (179-251)	435 (354-648)	<0.001
13 - 15	201 (176-247)	432 (293-524)	<0.001
≥16	199 (167-220)	349 (283-410)	<0.001
**Serum creatinine (Scr) (μmol/L)**			
0	70.0 (54.4-88.2)	75.5 (64.3-102.5)	0.147
1 - 3	72.5 (59.6-84.3)	76.0 (60.9-110.1)	0.337
4 - 6	70.3 (53.4-89.1)	76.4 (60.1-113.7)	0.176
7 - 9	62.6 (53.0-73.9)	78.5 (55.0-153.7)	0.026
10 - 12	63.8 (53.8-78.5)	72.1 (54.9-140.2)	0.172
13 - 15	66.6 (51.2-87.4)	71.6 (57.7-112.3)	0.272
≥16	61.1 (54.1-72.8)	63.1 (52.7-133.3)	0.491
**D-dimer (mg/L)**			
0	1.12 (0.43-5.42)	3.97 (0.78-8.00)	0.010

1 - 3	1.12 (0.43-3.76)	1.90 (0.62-7.81)	0.235
4 - 6	2.57 (0.85-6.25)	8.00 (5.38-8.00)	0.002
7 - 9	3.17 (0.67-4.89)	7.90 (5.29-8.00)	<0.001
10 - 12	1.75 (0.78-4.42)	7.18 (3.76-8.00)	<0.001
13 - 15	1.18 (0.49-2.79)	5.00 (2.54-8.00)	<0.001
≥16	1.02 (0.57-1.86)	4.23 (2.74-7.10)	<0.001
**Cardiac troponin I (cTnI) (µg/L)**			
0	9.9 (3.4-57.1)	24.1 (9.8-155.8)	0.006
1 - 3	17.0 (6.1-64.3)	30.1 (12.7-160.1)	0.254
4 - 6	22.2 (9.4-1093.6)	38.8 (14.1-328.4)	0.517
7 - 9	10.2 (6.4-45.4)	46.4 (9.9-295.2)	0.070
10 - 12	5.3 (2.7-16.0)	82.8 (10.7-270.4)	<0.001
13 - 15	4.4 (2.8-6.4)	43.6 (10.5-371.0)	<0.001
≥16	6.6 (4.8-19.6)	37.6 (21.7-183.1)	0.001
**Brain natriuretic peptide (BNP) (ng/L)**			
0	48.6 (21.1-119.5)	88.7 (39.6-192.0)	0.029
1 - 3	37.0 (21.1-68.9)	108.6 (42.7-297.1)	0.046
4 - 6	159.5 (44.0-749.2)	104.4 (53.4-168.9)	0.509
7 - 9	61.4 (45.9-150.1)	95.9 (51.3-286.4)	0.506
10 - 12	31.0 (17.1-46.7)	173.1 (71.7-420.0)	0.002
13 - 15	26.7 (10.0-50.2)	110.7 (63.9-349.0)	0.002
≥16	68.4 (16.6-271.5)	187.6 (92.7-435.4)	0.028

Table S3: Dynamic changes in cytokines and T-lymphocyte subsets analysis between patients with clinical stability and death events.

Time from admission (d)	Discharge (n=29)	Death event (n=48)	p value
**IL-2 (ng/L)**			
≤10	2.77 (2.56-2.88)	2.59 (2.43-3.26)	0.815
11 - 13	2.68 (2.53-2.87)	2.70 (2.60-2.88)	0.758
14 - 16	2.56 (2.43-2.63)	2.57 (2.36-2.97)	0.610
17 - 19	2.40 (2.28-2.80)	2.50 (2.29-4.00)	0.734
20 - 22	2.84 (2.51-3.59)	3.42 (2.43-3.85)	0.810
23 - 25	3.80 (3.06-4.19)	4.53 (4.40-5.47)	0.073
26 - 28	3.48 (3.39-4.36)	3.89 (2.95-5.22)	0.953
≥29	3.84 (3.27-4.48)	4.02 (3.90-4.85)	0.275
**IL-4 (ng/L)**			
≤10	2.18 (1.67-2.40)	2.16 (1.67-2.48)	0.868
11 - 13	1.90 (1.76-2.36)	1.90 (1.71-2.26)	0.918
14 - 16	1.85 (1.57-2.42)	2.10 (1.58-2.76)	0.544
17 - 19	1.90 (1.49-2.16)	1.81 (1.43-3.28)	0.945
20 - 22	2.42 (1.90-4.20)	2.48 (1.67-3.90)	0.885
23 - 25	4.45 (2.74-5.62)	4.26 (3.34-5.48)	0.927
26 - 28	3.00 (2.93-4.40)	3.59 (2.92-4.61)	0.859
≥29	3.31 (2.22-4.26)	3.76 (3.49-5.22)	0.351
**IL-6 (ng/L)**			
≤10	5.71 (4.38-18.30)	61.80 (21.00-907.53)	<0.001
11 - 13	4.97 (3.39-19.20)	48.46 (20.34-321.86)	0.002
14 - 16	14.79 (7.47-18.97)	22.27 (15.06-112.87)	0.039
17 - 19	7.88 (5.56-8.99)	109.38 (11.32-533.01)	0.014
20 - 22	6.32 (5.69-8.53)	128.30 (28.06-1308.50)	<0.001
23 - 25	9.51 (4.33-12.41)	223.81 (48.16-2460.85)	<0.001
26 - 28	8.78 (5.76-11.97)	682.38 (61.53-2602.46)	<0.001
≥29	6.18 (5.23-11.44)	2837.94 (217.95-4846.50)	<0.001
**IL-10 (ng/L)**			
≤10	3.46 (2.99-4.26)	5.41 (4.28-38.22)	0.010
11 - 13	3.75 (3.44-4.77)	4.97 (3.81-7.69)	0.142
14 - 16	4.29 (3.19-5.25)	5.91 (3.24-9.74)	0.162
17 - 19	4.15 (2.99-5.56)	5.51 (3.98-7.80)	0.365
20 - 22	3.70 (3.11-3.91)	5.95 (4.48-20.73)	0.049
23 - 25	5.88 (3.43-8.27)	8.15 (4.18-11.05)	0.412
26 - 28	5.88 (4.78-9.77)	14.01 (5.65-31.30)	0.129
≥29	6.97 (4.78-8.67)	46.70 (18.01-79.14)	0.003
**TNF-α (ng/L)**			
≤10	2.26 (1.93-2.57)	2.10 (1.92-3.20)	0.973
11 - 13	1.97 (1.77-2.27)	2.22 (1.84-2.73)	0.408

14 - 16	2.14 (1.88-2.64)	2.10 (1.68-2.71)	0.753
17 - 19	1.93 (1.88-2.09)	2.06 (1.65-3.05)	<0.001
20 - 22	1.89 (1.65-2.39)	2.74 (2.05-3.68)	0.080
23 - 25	3.98 (3.11-4.12)	4.34 (3.34-5.18)	0.412
26 - 28	2.30 (2.16-3.02)	3.34 (3.09-3.68)	0.099
≥29	3.29 (2.44-5.16)	3.46 (3.34-4.02)	0.393
**IFN-γ (ng/L)**			
≤10	1.98 (1.94-2.04)	1.82 (1.72-2.34)	0.441
11 - 13	1.92 (1.61-2.21)	1.92 (1.80-2.08)	<0.001
14 - 16	1.91 (1.76-2.25)	1.86 (1.47-2.46)	0.753
17 - 19	1.99 (1.96-2.04)	1.96 (1.39-3.19)	<0.001
20 - 22	2.22 (1.75-2.66)	2.52 (1.84-3.23)	0.665
23 - 25	4.17 (2.99-4.81)	4.14 (3.02-4.72)	0.788
26 - 28	3.45 (3.45-3.88)	3.25 (3.02-4.07)	0.768
≥29	2.92 (2.32-4.92)	3.40 (3.07-3.72)	0.757
**CD3+ T-lymphocyte ratio (%)**			
≤10	72.58 (60.93-79.97)	66.38 (60.04-74.90)	0.464
11 - 13	68.67 (53.91-80.11)	70.36 (60.11-72.65)	0.867
14 - 16	78.03 (70.44-82.48)	60.84 (56.42-66.48)	<0.001
17 - 19	66.91 (62.63-74.13)	68.35 (58.98-79.41)	0.959
20 - 22	76.18 (72.48-78.84)	63.62 (59.88-77.03)	0.119
23 - 25	78.21 (71.15-85.41)	67.48 (62.29-69.45)	0.015
26 - 28	84.45 (80.42-84.92)	61.95 (58.01-64.98)	0.001
≥29	72.98 (55.28-85.53)	58.06 (51.79-73.56)	0.336
**CD4+ T-lymphocyte ratio (%)**			
≤10	50.61 (36.22-57.53)	42.46 (36.89-51.01)	0.356
11 - 13	51.81 (33.82-54.41)	51.88 (40.63-56.36)	0.397
14 - 16	47.44 (45.23-50.28)	40.46 (34.86-47.15)	0.010
17 - 19	38.01 (36.22-45.32)	52.18 (44.53-58.76)	0.048
20 - 22	52.49 (51.35-55.89)	42.20 (34.36-56.14)	0.098
23 - 25	43.65 (29.41-50.25)	43.78 (32.94-49.66)	0.871
26 - 28	50.20 (32.79-52.27)	46.13 (34.59-46.58)	0.513
≥29	40.23 (30.52-43.50)	36.84 (30.52-54.20)	0.779
**CD8+ T-lymphocyte ratio (%)**			
≤10	17.02 (9.67-26.18)	16.88 (14.22-24.77)	0.621
11 - 13	18.29 (12.14-22.92)	15.35 (8.31-16.77)	0.336
14 - 16	23.74 (19.48-29.35)	17.83 (14.83-21.19)	0.007
17 - 19	23.57 (23.18-27.87)	13.81 (8.74-19.52)	0.004
20 - 22	17.60 (16.50-19.39)	18.44 (12.35-26.69)	0.612
23 - 25	28.47 (19.22-47.04)	17.48 (15.30-25.48)	0.350

26 - 28	27.73 (22.43-47.45)	16.20 (12.10-21.41)	0.013
≥29	29.21 (19.16-34.03)	15.52 (14.56-24.76)	0.054
**CD4+/CD8+ lymphocyte ratio**			
≤10	2.72 (1.75-5.67)	2.39 (1.67-3.67)	0.436
11 - 13	3.10 (1.48-3.89)	3.58 (2.67-7.46)	0.189
14 - 16	2.01 (1.65-2.855)	2.17 (1.88-2.74)	0.113
17 - 19	1.61 (1.56-1.63)	4.175 (2.74-5.49)	0.004
20 - 22	3.11 (2.71-3.25)	2.7 (1.45-3.38)	0.349
23 - 25	1.64 (0.945-2.64)	2.44 (1.31-3.2)	0.477
26 - 28	1.88 (0.69-2.52)	2.775 (1.79-3.82)	0.206
≥29	1.42 (1.10-2.00)	1.87 (1.55-2.93)	0.152
